# Outbreak investigation of reemerging chikungunya virus in Malaysia in 2019–2020

**DOI:** 10.1186/s12879-026-13793-7

**Published:** 2026-06-11

**Authors:** Yik-Zheng Lim, Boon-Teong Teoh, Chee-Sieng Khor, Nadrah-Arfizah Arifin, Kim-Kee Tan, Juraina Abd-Jamil, Noor-Syahida Azizan, Che-Norainon Yaacob, Nurul-Farhanah Hanuar, Sarah Affendi, Jefree Johari, Jia-Yi Tan, Wei Yin Vinnie-Siow, Sing-Sin Sam, Van-Lun Low, Sazaly AbuBakar

**Affiliations:** 1 Tropical Infectious Diseases Research and Education Centre (TIDREC), Higher Institution Centre of Excellence (HICoE), Universiti Malaya, Kuala Lumpur, Malaysia; 2 Batang Padang District Health Office, Ministry of Health Malaysia, Perak, Malaysia

**Keywords:** Chikungunya virus, Disease outbreaks, Malaysia, Phylogeny, Genotype

## Abstract

**Background:**

Chikungunya virus (CHIKV) is a mosquito-borne pathogen that imposes a substantial public health burden in tropical regions. After a decade of sporadic transmission, Malaysia experienced an alarming resurgence of CHIKV cases in 2019. This study investigated the reemergence of CHIKV in Malaysia following this prolonged period of sporadic transmission.

**Methods:**

An outbreak investigation was conducted in two phases across three hotspots in Perak, Malaysia (Kampung Baru Air Kuning, Bidor, and Sungkai) between October 2019 and February 2020. Blood samples were collected from 78 subjects and were tested by real-time reverse transcription polymerase chain reaction (RT-PCR) and enzyme-linked immunosorbent assays (ELISA). CHIKV was isolated and subjected to whole-genome sequencing. The resulting sequences were then analysed using phylogenetic, comparative sequence and selection pressure analyses.

**Results:**

A high CHIKV positivity rate of 69.2% (54/78) was observed in this study, with a 9.3% (5/54) rate of dengue virus (DENV) coinfection. Phylogenetic analysis revealed that the CHIKV isolates from this study belonged to the East/Central/South African-Indian Ocean Lineage (ECSA-IOL) genotype and clustered with recent outbreak isolates from Thailand, China, Myanmar, Bangladesh, Italy, Pakistan, and India during 2015–2019, rather than the Malaysia isolate from the previous 2009 outbreak. Malaysia 2019 and Thailand 2018 isolates shared relatively higher nucleotide similarity (99.78–99.89%). The reemerging CHIKV in Malaysia harboured E1-226 A, E1-K211E, and E2-V264A which enhance CHIKV fitness in *Aedes aegypti*, as well as nsP3-R524Opal that is associated with increased virulence. These mutations were consistent with globally circulating epidemic strains during 2015–2019, highlighting vector adaptation and disease severity.

**Conclusions:**

The reemerging ECSA-IOL strain in Malaysia was genetically distinct from the Malaysia 2009 outbreak strain but closely related to the globally circulating epidemic strains during 2015–2019. Collectively, it was likely introduced from neighbouring Thailand through cross-border transmission.

**Supplementary Information:**

The online version contains supplementary material available at 10.1186/s12879-026-13793-7.

## Background

Chikungunya virus (CHIKV) belongs to *Alphavirus* genus in the *Togaviridae* family [1]. This arthropod-borne virus is transmitted to humans by *Aedes aegypti* and *Ae. albopictus* mosquitoes [1, 2]. Its viral genome is a single-stranded, positive-sense RNA of approximately 11.8 kb, comprising two open reading frames (ORFs): 1) 5’ ORF encodes four non-structural proteins (nsP1, nsP2, nsP3 and nsP4), and 2) 3’ ORF encodes five structural proteins, including the capsid protein (C), envelope proteins (E1, E2 and E3) and the accessory protein (6 K) [3, 4].

Historical evidence suggests CHIKV originated from Central and Western Africa [5]. CHIKV might have originated around 300 to 500 years ago but the infection was frequently misdiagnosed as other infectious diseases with similar clinical manifestations due to lack of diagnostic tools in the pre-virology era [6]. CHIKV was first isolated in 1953 from Newala district of Tanzania during the first documented CHIKV outbreak (1952–1953) [7, 8]. Since then, CHIKV has caused recurrent outbreaks worldwide over recent decades [9–11]. Most recently, an outbreak of chikungunya fever was reported in Guangdong Province, China in 2025 [12, 13].

The phylogeny of CHIKV comprises three distinct genotypes: West African, Asian and East/Central/South African (ECSA) [14, 15]. ECSA and Asian strains have given rise to epidemic sub-lineages like Indian Ocean Lineage (IOL) and Caribbean Outbreak Lineage (COL), respectively [16, 17]. Prior to 2019, there were four documented CHIKV outbreaks in Malaysia: (1) Port Klang, Selangor in 1998 (Asian genotype) [18], (2) Bagan Panchor, Perak in March 2006 (Asian genotype) [19], (3) Ipoh, Perak in December 2006 (ECSA-IOL genotype) [20], and (4) nationwide outbreak during 2008–2009 (ECSA-IOL genotype) [21]. Following a decade of sporadic transmission, the Ministry of Health Malaysia reported an alarming resurgence in CHIKV cases in Malaysia, particularly in Perak state, from only one case (1.15%, 1/87) in 2018 to 582 cases (62.2%, 582/935) in 2019 [22]. A comprehensive investigation on the reemerging CHIKV outbreak in Malaysia in 2019 after a 10-year period of sporadic transmission was therefore warranted. Additionally, gaining insight into the origin of the responsible CHIKV strain in the reemerging outbreak was important for understanding the current epidemiology of CHIKV.

## Methods

### Sample collection and laboratory diagnosis

**Fig. 1:**
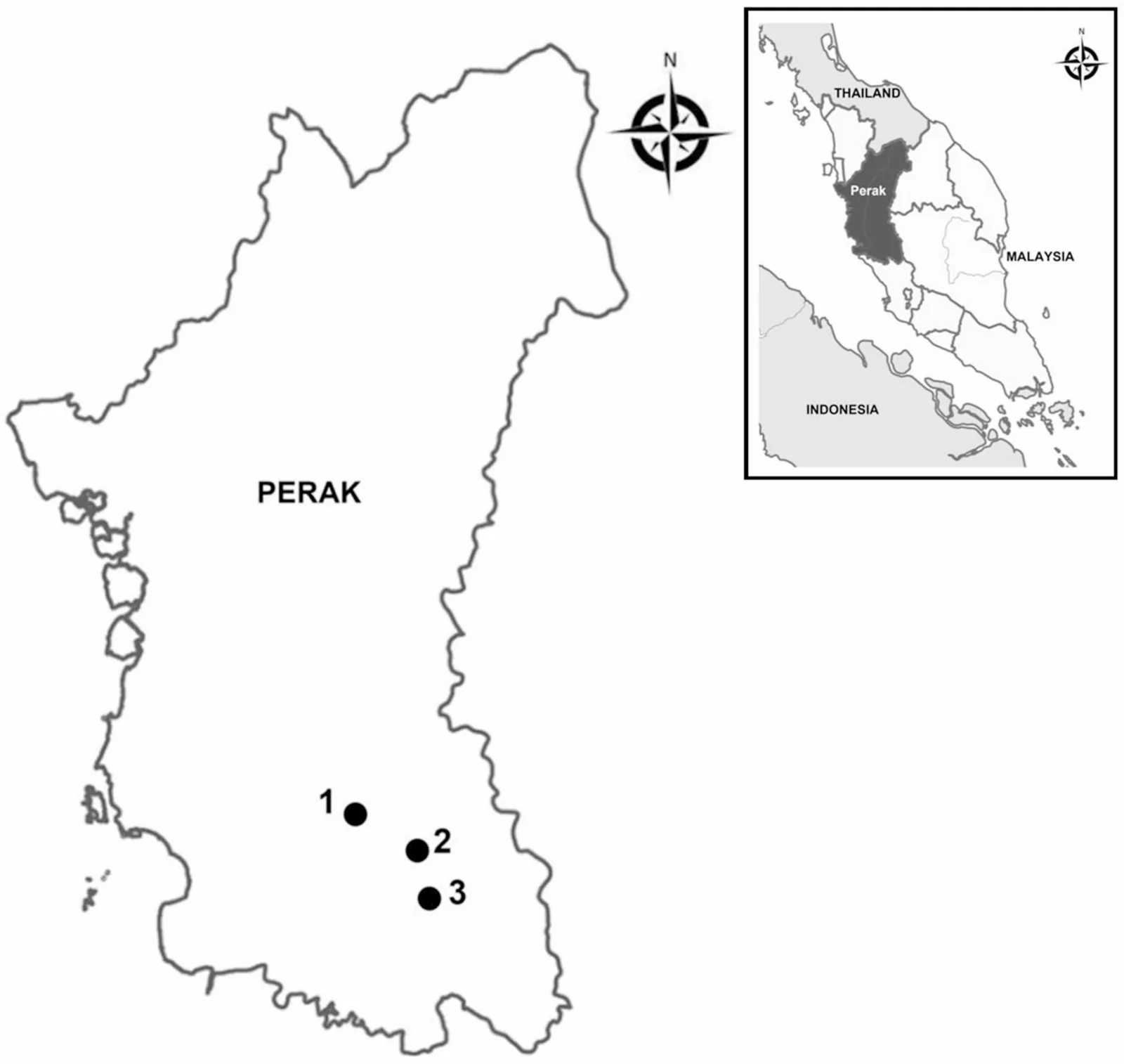
Study sites in Perak state, Malaysia in 2019–2020. The outbreak investigation was conducted in two phases: (1) first phase in October 2019 in Kampung Baru Air Kuning (site 1), and (2) second phase in February 2020 in Bidor (site 2) and Sungkai (site 3). Inset map shows location of Perak in Malaysia (black shading)

On October 16, 2019, Tropical Infectious Diseases Research and Education Centre (TIDREC) was notified by a local general practitioner regarding an increase in the number of patients presenting with chikungunya-like illness in Kampung Baru Air Kuning, Perak state, Malaysia. This initiated our outbreak investigation at the suspected outbreak hotspots in Perak (Fig. 1). Following World Health Organization (WHO) guidelines, a suspected case is defined as any person with acute fever and severe joint pain; a probable case is suspected case with recent residence in or travel to epidemic area within 15 days before symptom onset; and a confirmed case is a suspected case which is laboratory-confirmed by virus isolation, reverse transcription polymerase chain reaction (RT-PCR) or enzyme-linked immunosorbent assay (ELISA) [23].

Approximately 5 mL of venous blood was collected into serum separator tubes (BD Vacutainer, USA) from: (1) patients by the physicians when seeking treatment at local clinics, and (2) residents by certified phlebotomists during a health screening provided by Batang Padang District Health Office, Ministry of Health Malaysia. The samples were then sent to a MS ISO/IEC 17,025-accredited laboratory at TIDREC for diagnosis. Demographic and clinical information, including sex, age, days post-onset (dpo) and clinical manifestations, were recorded for all subjects for further analysis.

**Fig. 2:**
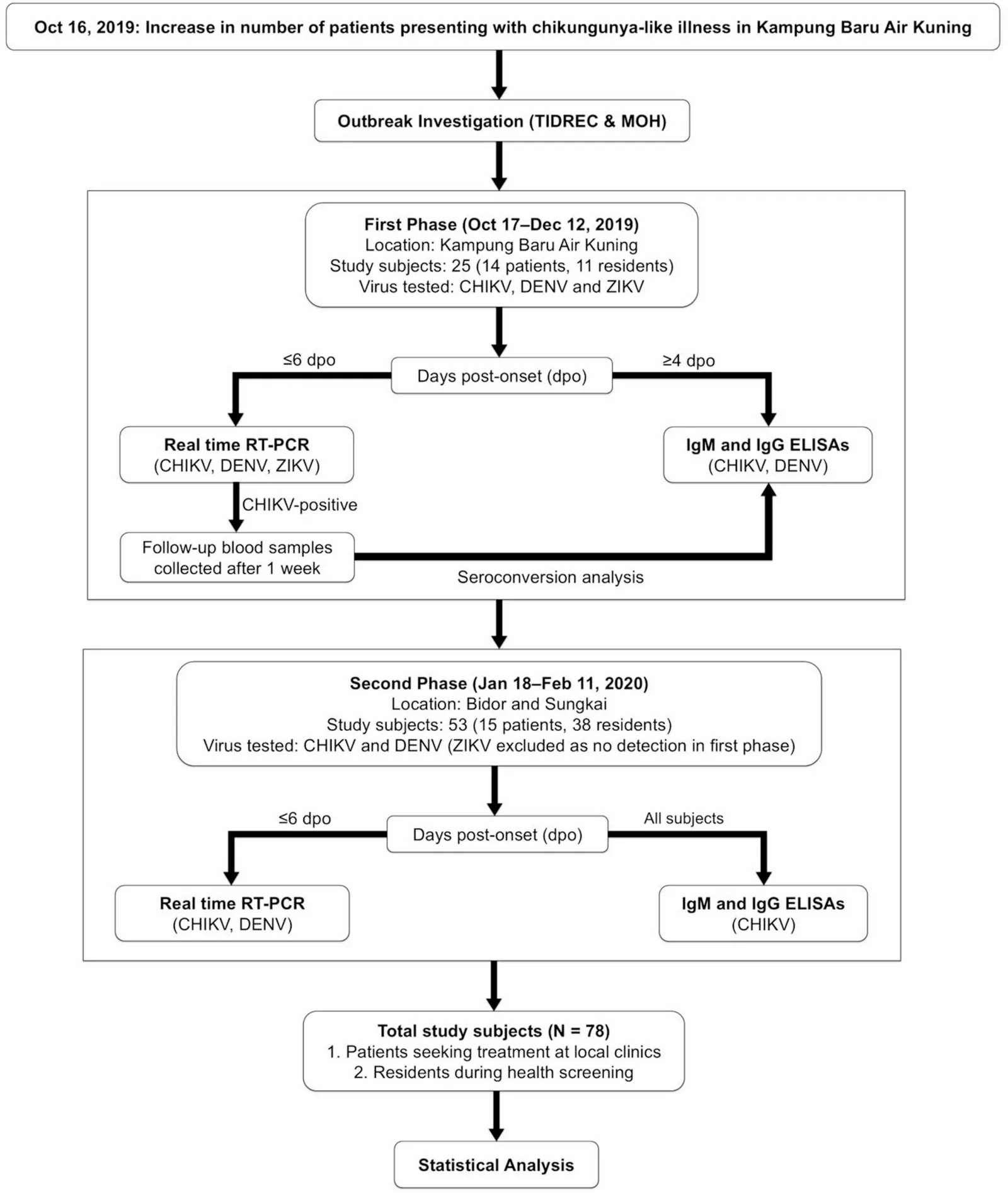
Workflow of outbreak investigation in Perak, Malaysia in 2019–2020

Our outbreak investigation comprised two phases. In the first phase (October 17 to December 12, 2019), blood samples were collected from 25 subjects (14 patients and 11 residents) in Kampung Baru Air Kuning. They were tested for CHIKV, dengue virus (DENV) and Zika virus (ZIKV) by real-time RT-PCR or ELISA according to the timing of sample collection relative to dpo. Subjects with ≤ 6 dpo were tested by in-house real-time RT-PCR for CHIKV, DENV and ZIKV [24, 25], while those with ≥ 4 dpo were tested by IgM and IgG ELISAs (Euroimmun, Germany) for CHIKV and DENV [26, 27]. The virus positivity was defined as a positive result in either real-time RT-PCR, IgM ELISA or both, indicating active infection among study subjects. Seroconversion analysis was performed on CHIKV-specific real-time RT-PCR-positive patients by collecting a follow-up blood sample one week later and testing it with anti-CHIKV IgM and IgG ELISAs. Following that, the investigation was expanded to Bidor and Sungkai which are adjacent to Air Kuning, to track the virus spread. In the second phase (January 18 to February 11, 2020), blood samples were collected from 53 subjects (15 patients and 38 residents). ZIKV testing was excluded due to its absence in the first phase. Subjects with ≤ 6 dpo were tested by real-time RT-PCR for CHIKV and DENV. All subjects, regardless of dpo, were tested by anti-CHIKV IgM and IgG ELISAs. The workflow of the outbreak investigation is presented in Fig. 2.

### Real-time RT-PCR

The in-house real-time RT-PCR assays were performed as previously described [24, 25]. The SensiFAST Probe Hi-ROX One-Step Kit (Meridian Bioscience, USA) was used for all real-time RT-PCR reactions. Each reaction was performed in a final volume of 15 µL containing 7.5 µL of 2× SensiFAST Probe Hi-ROX One-Step Mix, 0.6 µL of 10 µM forward primer, 0.6 µL of 10 µM reverse primer, 0.15 µL of 10 µM probe, 0.15 µL of reverse transcriptase, 0.3 µL of RiboSafe RNase inhibitor, 3.7 µL of nuclease-free water, and 2 µL of RNA template. The thermocycling conditions consisted of reverse transcription at 45 °C for 10 min, polymerase activation at 95 °C for 2 min, followed by 40 cycles of denaturation at 95 °C for 5 s, annealing at 55 °C for 10 s, and extension at 60 °C for 20 s. All reactions were performed using StepOnePlus Real-Time PCR System (Applied Biosystems, USA). Raw data were analysed using StepOne Software version 2.2.1 (Applied Biosystems, USA), and samples with a cycle threshold (Ct) value of < 40 were considered positive. The primers and probes used are listed in Supplementary Table 1.

### Statistical analysis

Bivariate analysis was performed using SPSS Statistics version 26, with *p* < 0.05 considered statistically significant. First, the associations between CHIKV positivity and demographic characteristics (sex and age) were assessed among all study subjects. Second, among CHIKV-positive subjects, the associations of clinical manifestations (fever, joint pain, and rash) with sex, age and dpo were assessed. Age, dpo, and clinical manifestation data were not recorded for all study subjects. Analyses for each variable were restricted to subjects with available data only: sex (*n* = 78), age group (*n* = 75), dpo (*n* = 53) and clinical manifestation data (*n* = 53). For all analyses, Pearson’s Chi-square test was applied when all expected cell counts were ≥ 5, and Fisher’s exact test was applied when one or more expected cell counts were < 5.

### Virus isolation and whole-genome sequencing

*Aedes albopictus* larval cells (C6/36) were cultured in the Eagle’s Minimum Essential Medium (EMEM) supplemented with 10% heat-inactivated fetal bovine serum (FBS). The cells were maintained at 28 °C in a 3% CO_2_ atmosphere and subpassaged upon reaching confluence. For virus isolation, the cells were seeded at 2 × 10^5^ cells/well in a 24-well plate containing EMEM supplemented with 2% FBS and incubated overnight. The serum samples that tested positive by CHIKV-specific real time RT-PCR were used for cell inoculation. Viral RNAs were extracted from harvested viruses using QIAamp Viral RNA Mini Kit (Qiagen, USA). They were then subjected to Ion Torrent whole-genome sequencing service provided by TIDREC as previously described [28]. The resulting sequencing raw reads were assembled using Genomic Short-read Nucleotide Alignment Program (GSNAP) algorithms implemented in Sequencher 5.2.2.

### Phylogenetic analysis

CHIKV complete coding sequences (CDS) were retrieved from National Library of Medicine (NCBI) GenBank and screened to exclude sequences containing undetermined nucleotides. After excluding sequences with undetermined nucleotides, 730 CHIKV sequences were aligned using GeneDoc version 2.7. Following that, preliminary neighbour joining (NJ) phylogenetic analysis was performed using Molecular Evolutionary Genetics Analysis (MEGA) version 10.2.2 to reduce the computational burden of subsequent maximum likelihood (ML) phylogenetic analysis. From this NJ tree, representative sequences were selected from each monophyletic group to ensure coverage of the genetic diversity across different geographical regions and temporal periods. These representative sequences were aligned with the Malaysia 2019 outbreak isolates from this study using ClustalX version 2.1. A ML tree was then constructed using MEGA version 10.2.2.

### Comparative sequence analysis

The nucleotide similarities at CDS region between representative CHIKV sequences and Malaysia 2019 outbreak isolates were determined using Basic Local Alignment Search Tool (BLAST) (https://blast.ncbi.nlm.nih.gov/Blast.cgi). Moreover, the amino acid substitutions between Malaysia 2019 and 2009 outbreak isolates were identified using GeneDoc version 2.7. The types of non-synonymous substitutions (conservative, semi-conservative and non-conservative) were subsequently determined using ClustalX version 2.1.

### Selection pressure analysis

Selection pressure was analysed via the Datamonkey adaptive evolution server (http://www.datamonkey.org). Two datasets: (1) CHIKV non-structural polyprotein nucleotide sequences (*n* = 92) and (2) structural polyprotein nucleotide sequences (*n* = 92) were analysed. The selection pressure at individual codon sites was determined using Single-Likelihood Ancestor Counting (SLAC), Fixed Effects Likelihood (FEL), Mixed Effects Model of Evolution (MEME) and Fast Unconstrained Bayesian Approximation (FUBAR) methods. A codon was considered a positively or negatively selected site when detected by at least one of the following methods: FEL (*p* ≤ 0.05), SLAC (*p* ≤ 0.05); MEME (*p* ≤ 0.05); FUBAR (*p* ≤ 0.05).

## Results

### CHIKV outbreak investigation (2019–2020)

**Fig. 3:**
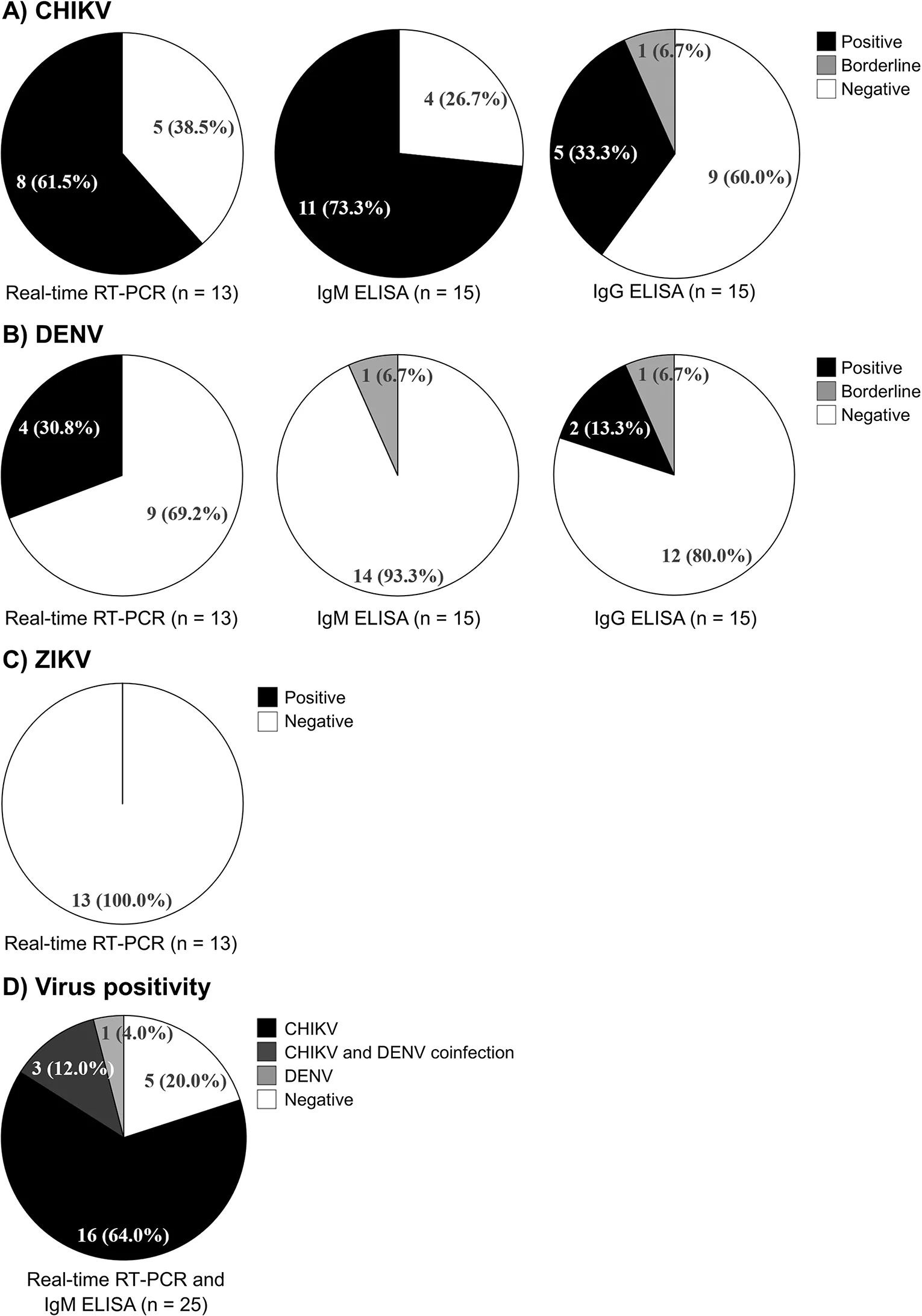
First phase of outbreak investigation in Malaysia, October 17 to December 12, 2019. Twenty-five study subjects were tested according to days post-onset (dpo): 13 (≤ 6 dpo) by real-time RT-PCR for CHIKV, DENV and ZIKV, and 15 (≥ 4 dpo) by ELISA for CHIKV and DENV. Three subjects with 4–6 dpo were tested by both methods. Borderline ELISA results were interpreted according to the Euroimmun kit manual. The virus positivity was defined as a positive result in either real-time RT-PCR, IgM ELISA or both, indicating active infection. CHIKV, chikungunya virus; DENV, dengue virus; ZIKV, Zika virus; RT-PCR, reverse transcription polymerase chain reaction; ELISA, enzyme-linked immunosorbent assay; IgM, immunoglobulin M; IgG, immunoglobulin G

**Fig. 4:**
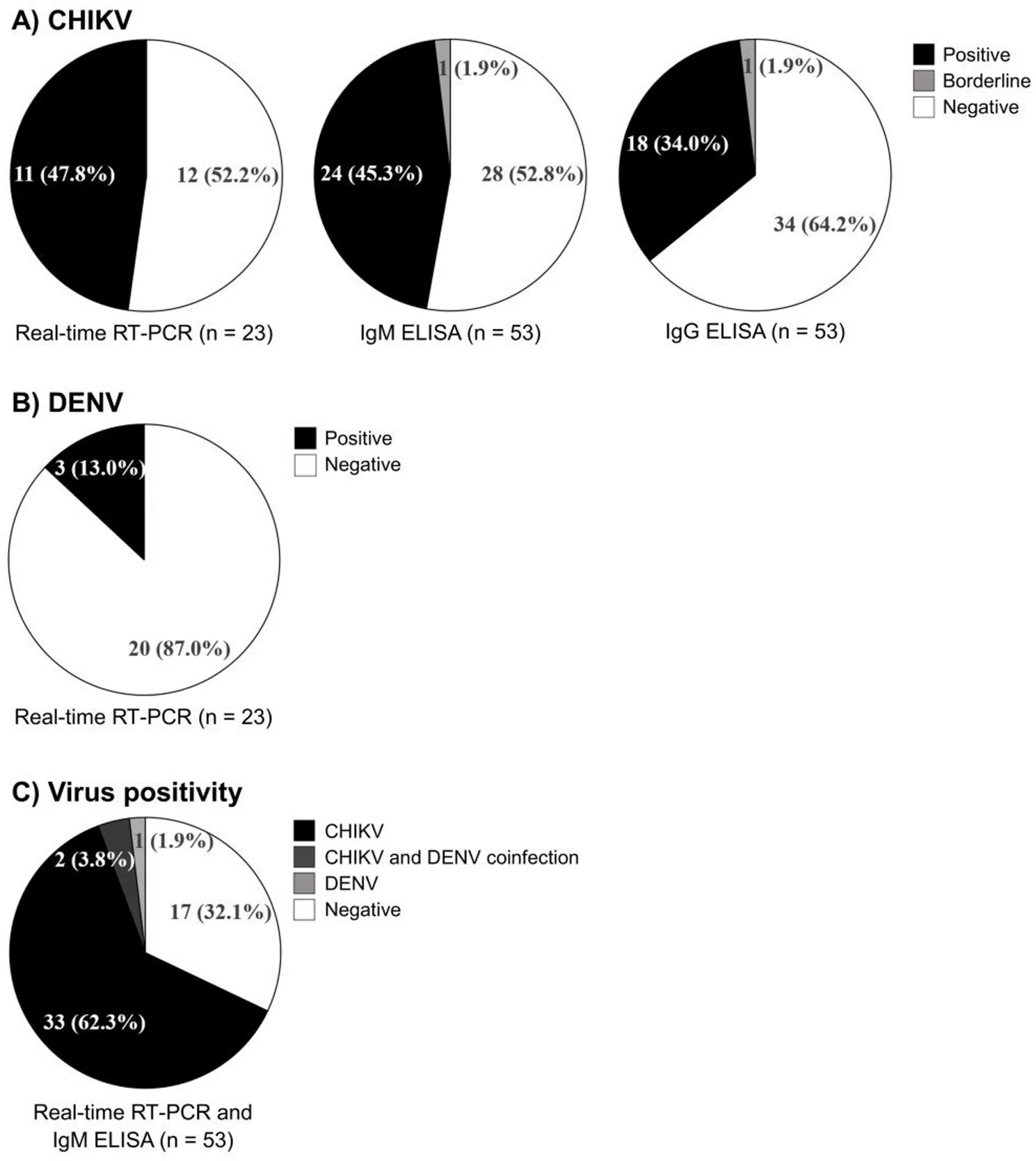
Second phase of outbreak investigation in Malaysia, January 18 to February 11, 2020. Fifty-three study subjects were tested according to days post-onset (dpo): 23 (≤ 6 dpo) by real-time RT-PCR for CHIKV and DENV, and all subjects by ELISA for CHIKV, regardless of dpo. Borderline ELISA results were interpreted according to the Euroimmun kit manual. The virus positivity was defined as a positive result in either real-time RT-PCR, IgM ELISA or both, indicating active infection. CHIKV, chikungunya virus; DENV, dengue virus; RT-PCR, reverse transcription polymerase chain reaction; ELISA, enzyme-linked immunosorbent assay; IgM, immunoglobulin M; IgG, immunoglobulin G

A total of 78 subjects were involved in this study, with 31 (39.7%) males and 47 (60.3%) females. Their ages ranged from 6 to 90 years, with a median age of 57 years (interquartile range [IQR]: 46–66). The first phase of outbreak investigation (Fig. 3) revealed a CHIKV positivity rate of 76.0% (19/25) in Kampung Baru Air Kuning, with 15.8% (3/19) coinfection with DENV. No ZIKV infection was detected. Analysis of four convalescent paired samples confirmed anti-CHIKV IgM seroconversion. Further investigation in the adjacent areas (Fig. 4) showed a CHIKV positivity rate of 66.0% (35/53) in Bidor and Sungkai, with 5.7% (2/35) coinfection with DENV. Overall, CHIKV infection was detected in 69.2% of subjects (54/78) across the three study sites in Perak, with 9.3% (5/54) coinfection with DENV. The CHIKV seropositivity rate was 52.9% (36/68), with 97.2% (35/36) of seropositive samples being positive for anti-CHIKV IgM (Table 1).

**Table 1:** Comparison of anti-CHIKV IgM and IgG ELISA results among study subjects in Malaysia, 2019–2020*

IgM ELISA, no. (%)	IgG ELISA, no. (%)			Total no. (%)
	Positive	Borderline	Negative	
Positive	22 (32.4)	2 (2.9)	11 (16.2)	35 (51.5)
Borderline	0 (0)	0 (0)	1 (1.5)	1 (1.5)
Negative	1 (1.5)	0 (0)	31 (45.6)	32 (47.1)
Total no. (%)	23 (33.8)	2 (2.9)	43 (63.2)	68 (100.0)

*Sixty-eight subjects were tested by ELISA: 15 subjects with ≥ 4 days post-onset from the first phase and 53 subjects from the second phase of outbreak investigation. CHIKV, chikungunya virus; IgM, immunoglobulin M; IgG, immunoglobulin G; ELISA, enzyme-linked immunosorbent assay

### Statistical analysis

The results are presented in Table 2. CHIKV positivity did not differ significantly between males and females (*p* = 0.943), nor across age groups (*p* = 0.865). Among CHIKV-positive subjects, no significant associations were observed between sex and clinical manifestations (fever: *p* = 0.221; joint pain: *p* = 0.215; rash: *p* = 0.735), or between age group and clinical manifestations (fever: *p* = 0.842; joint pain: *p* = 0.345; rash: *p* = 0.060). With respect to dpo, fever and joint pain did not differ significantly between CHIKV-positive subjects with ≤ 3 dpo and ≥ 4 dpo (fever: *p* = 0.522; joint pain: *p* = 1.000). In contrast, rash was significantly more prevalent in CHIKV-positive subjects with ≥ 4 dpo (10/22, 45.5%) than those with ≤ 3 dpo (2/17, 11.8%) (*p* = 0.024).

**Table 2:** Bivariate analysis of CHIKV positivity by sex and age group among all study subjects, and clinical manifestations by sex, age group, and days post-onset among CHIKV-positive subjects*

Variable	No. subjects (%)	No. CHIKV-positive subjects (%)	No. CHIKV-positive subjects with clinical manifestation (%)		
			Fever	Joint pain	Rash
Sex					
Male	31 (39.7)	22 (71.0)	12 (66.7)	17 (94.4)	4 (22.2)
Female	47 (60.3)	33 (70.2)	13 (48.1)	21 (77.8)	8 (29.6)
* p* value^†^	NA	0.943	0.221	0.215	0.735
Age group					
≤ 10	2 (2.7)	2 (100.0)	2 (100.0)	2 (100.0)	1 (50.0)
1–20	3 (4.0)	2 (66.7)	0 (0)	0 (0)	0 (0)
21–30	4 (5.3)	3 (75.0)	1 (33.3)	3 (100.0)	2 (66.7)
31–40	5 (6.7)	4 (80.0)	3 (75.0)	4 (100.0)	1 (25.0)
41–50	9 (12.0)	6 (66.7)	3 (60.0)	4 (80.0)	2 (40.0)
51–60	22 (29.3)	12 (54.5)	8 (66.7)	9 (75.0)	6 (50.0)
61–70	16 (21.3)	12 (75.0)	4 (44.4)	8 (88.9)	0 (0)
71–80	11 (14.7)	9 (81.8)	3 (60.0)	5 (100.0)	0 (0)
≥ 81	3 (4.0)	2 (66.7)	1 (50.0)	1 (50.0)	0 (0)
* p* value^**†**^	NA	2 (100.0)	0.842	0.345	0.060
Dpo					
≤ 3	20 (37.7)	18 (45.0)	11 (64.7)	16 (94.1)	2 (11.8)
≥ 4	33 (62.3)	22 (55.0)	12 (54.5)	20 (90.9)	10 (45.5)
* p* value^**†**^	NA	NA	0.522	1.000	**0.024**

*Age, dpo, and clinical manifestation data were not recorded for all study subjects. Analyses for each variable were restricted to subjects with available data: sex (*n* = 78), age group (*n* = 75), dpo (*n* = 53) and clinical manifestation data (*n* = 53). CHIKV-positive subjects with unrecorded clinical manifestation data were excluded from the corresponding analyses. ^**†**^Pearson’s Chi-square test was applied when all expected cell counts were ≥ 5; Fisher’s exact test was applied when one or more expected cell counts were < 5. dpo, days post-onset; NA, not applicable

### Phylogenetic analysis

**Fig. 5:**
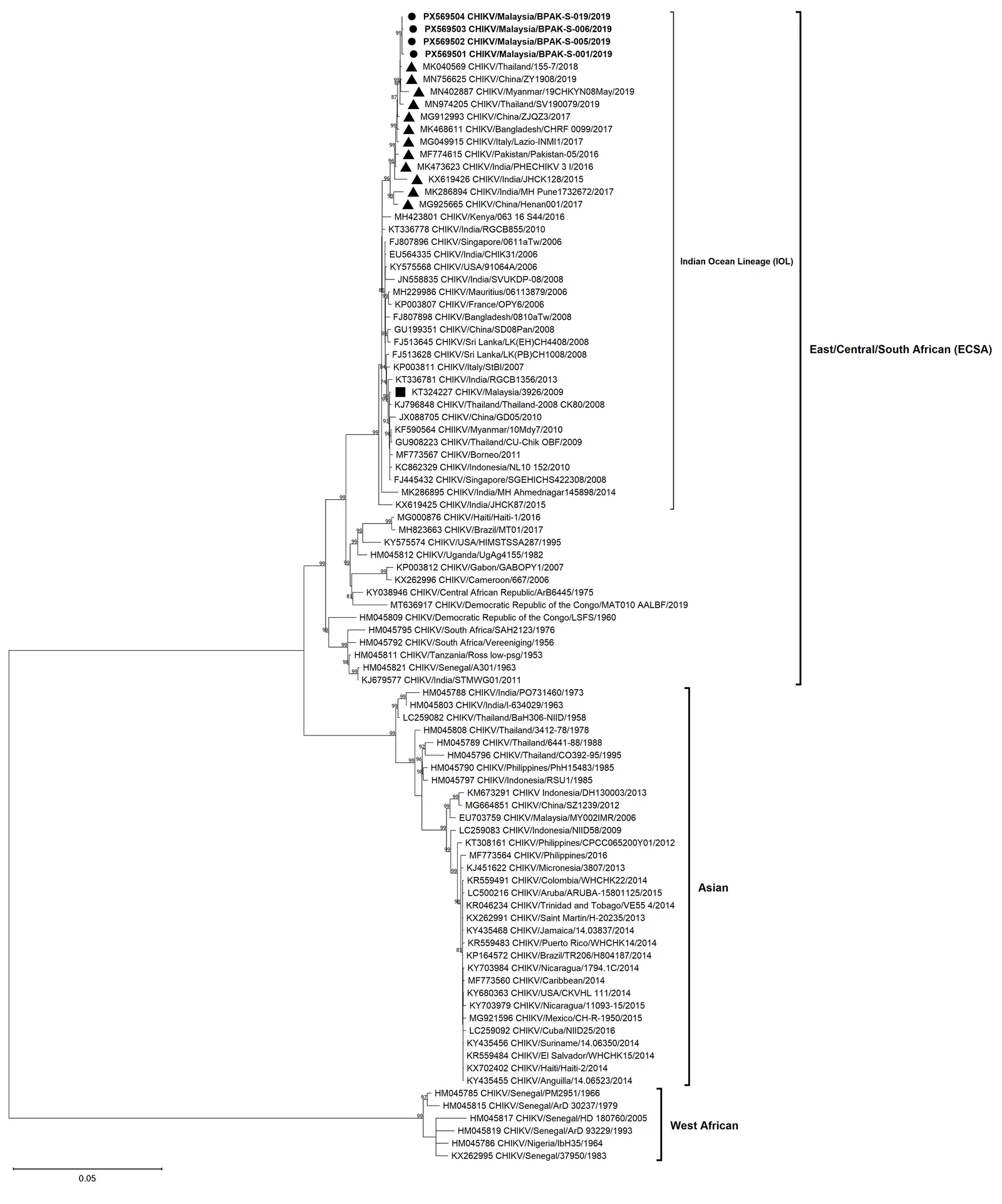
Phylogenetic tree of chikungunya virus whole-genome. The phylogeny was inferred using the maximum likelihood method and General Time Reversible with gamma distribution and invariant sites (GTR + G_5_+I) model. Bootstrap values from 1,000 replicates ≥ 70% are shown at the nodes. Scale bar indicates number of substitutions per site. Circles denote Malaysia 2019 outbreak isolates from this study; square denotes previous 2009 outbreak isolate from Malaysia; triangles denote recent outbreak isolates (2015–2019) that clustered with Malaysia 2019 outbreak isolates

The ML phylogenetic tree (Fig. 5) was constructed using four Malaysia 2019 outbreak isolates from this study and 88 representative CHIKV sequences retrieved from GenBank. The Malaysia 2019 outbreak isolates belonged to Indian Ocean Lineage (IOL) within the ECSA genotype. They clustered with recent outbreak isolates from Thailand, China, Myanmar, Bangladesh, Italy, Pakistan, and India during 2015–2019, forming a distinct branch separate from the previous 2009 outbreak isolate from Malaysia.

### Molecular characterization of reemerging CHIKV

The Malaysia 2019 outbreak isolates from this study were highly similar to one another (99.90–100%). Their pairwise nucleotide identity with the Malaysia 2009 outbreak isolate was slightly lower (99.02–99.06%) compared with the similarity to recent outbreak isolates from other countries during 2015–2019 (99.15–99.89%). Among these recent outbreak isolates, the highest nucleotide similarity was observed with the China 2019 and Thailand 2018 outbreak isolates (99.82–99.89%). A total of 23 amino acid substitutions were identified in Malaysia 2019 outbreak isolates compared with Malaysia 2009 outbreak isolate (Table 3), with most changes in structural proteins, particularly in E2 (1.18%) and C (1.15%). The positively and negatively selected sites identified across the CHIKV protein-coding regions are shown in Tables 4 and 5, respectively.

**Table 3:** Amino acid differences comparison between Malaysia 2019 and 2009 outbreak isolates*

Accession no.	Country	Year	nsp1	nsp2					nsp3				nsp4		C			E2					E1		
			108	130	145	495	539	793	258	372	441	524	55	85	8	27	73	146	205	252	264	397	211	226	317
KT324227	Malaysia	2009	L	H	E	N	S	V	T	D	T	Opal	S	R	A	I	K	Q	G	Q	V	I	K	V	I
PX569501	Malaysia	2019	·	Y	D	S	L	A	K	E	A	R	N	G	T	T	R	R	S	K	A	V	E	A	V
PX569502	Malaysia	2019	·	Y	D	S	L	A	K	E	A	·	N	G	T	·	R	·	S	K	A	·	E	A	V
PX569503	Malaysia	2019	R	Y	D	S	L	A	K	E	A	R	N	G	T	·	R	·	S	K	A	·	E	A	V
PX569504	Malaysia	2019	·	Y	D	S	L	A	K	E	A	·	N	G	T	·	R	·	S	K	A	·	E	A	V
KX619426	India	2015	·	Y	D	·	L	·	K	·	·	·	N	G	T	·	·	·	·	K	A	·	E	A	V
MK473623	India	2016	·	Y	D	·	L	·	K	·	·	·	N	G	T	·	·	·	·	K	A	·	E	A	V
MK286894	India	2017	·	Y	·	·	L	·	K	·	·	R	·	G	T	·	·	·	·	K	A	·	E	A	V
MF774615	Pakistan	2016	·	Y	D	·	L	·	K	·	·	·	N	G	T	·	·	·	·	K	A	·	E	A	V
MG049915	Italy	2017	·	Y	D	·	L	·	K	E	·	·	N	G	T	·	·	·	S	K	A	·	E	A	V
MK468611	Bangladesh	2017	·	Y	D	·	L	·	K	E	·	·	N	G	T	·	·	·	S	K	A	·	E	A	V
MG912993	China	2017	·	Y	D	·	L	·	K	E	·	·	N	G	T	·	·	R	S	K	A	·	E	A	V
MG925665	China	2017	·	Y	·	·	L	·	K	.	·	·	.	G	T	·	·	·	·	K	A	·	E	A	V
MN756625	China	2019	·	Y	D	S	L	A	K	E	·	·	N	G	T	·	R	·	S	K	A	·	E	A	V
MN974205	Thailand	2018	·	Y	D	S	L	A	K	E	·	·	N	G	T	·	R	·	S	K	A	·	E	A	V
MK040569	Thailand	2019	·	Y	D	S	L	A	K	E	·	R	N	G	T	·	R	·	S	K	A	·	E	A	V
MN402887	Myanmar	2019	·	Y	D	S	L	A	K	E	·	R	N	G	T	·	R	·	S	K	A	·	E	A	V
**Non-synonymous substitution** ^**†**^			/	:	:	.	/	.	.	:	:	/	.	/	:	/	:	:	.	:	.	:	:	.	:
**Selection pressure** ^**‡**^			n	–	n	n	n	n	+	+	n	n	n	n	n	n	n	–	–	–	–	–	–	–	–

*Twenty-three amino acid substitutions were identified in Malaysia 2019 outbreak isolates compared with Malaysia 2009 outbreak isolate. Residues of recent outbreak isolates from other countries (2015–2019) at these 23 positions are shown for comparison. Dot (·) indicates residue identical to Malaysia 2009 outbreak isolate. Opal at nsP3 position 524 denotes an opal stop codon (TGA). ^†^Non-synonymous substitutions are denoted as non-conservative (/), conservative (:), and semi-conservative (.). ^‡^Selection pressures are denoted as positive (+), negative (–) and neutral or invariable (n)

**Table 4:** Positively selected sites found in chikungunya virus genes*

Gene	Position	FEL	SLAC	MEME	FUBAR
		*p* value	*p* value	*p* value	posterior probability
nsP1	256	0.2282	0.464	**0.05**	0.522
	376	0.3665	0.771	**0.03**	0.057
	428	0.2697	0.445	**0.01**	0.485
nsP2	59	0.4024	0.656	**0.04**	0.387
	122	0.5333	0.767	**0.01**	0.079
	149	0.8700	0.734	**0**	0.210
	352	0.8857	0.741	**0**	0.273
	457	0.5626	0.841	**0**	0.353
	599	0.0731	0.198	**0.05**	0.726
	702	0.2419	0.444	**0.03**	0.496
nsP3	258	0.4350	0.737	**0.05**	0.374
	328	0.1745	0.335	**0.03**	0.565
	331	**0.0296**	0.088	**0.04**	0.916
	352	0.1133	0.327	**0.04**	0.701
	372	0.1682	0.488	**0.02**	0.573
nsP4	81	**0.0303**	0.100	**0**	**0.969**
	400	0.5687	0.744	**0.04**	0.083
	592	0.1962	0.963	**0**	0.038
C	94	0.4189	0.625	**0.01**	0.407
E3	33	0.0640	0.138	0.09	**0.960**
E2	57	0.2327	0.473	**0**	0.593
	241	0.1344	0.888	**0.01**	0.002
	242	0.5093	0.889	**0.02**	0.150
	246	0.1111	0.445	**0.04**	0.678
	248	0.8198	0.595	**0.05**	0.377
6 K	47	**0.0459**	0.296	**0.05**	0.818
E1	ND	ND	ND	ND	ND

*A site was considered under positive selection when detected by at least one of the following methods: FEL, SLAC or MEME at *p* ≤ 0.05, or FUBAR at posterior probability ≥ 0.95. Boldface indicates statistical significance: *p* ≤ 0.05 for SLAC, FEL, and MEME, and posterior probability ≥ 0.95 for FUBAR. SLAC, Single-Likelihood Ancestor Counting; FEL, Fixed Effects Likelihood; FUBAR, Fast Unconstrained Bayesian Approximation; MEME, Mixed Effects Model of Evolution; ND, not detected

**Table 5:** Negatively selected sites found in chikungunya virus genes*

Genes	No. negatively selected sites			No. negatively selected sites/Total no. sites (%)
	FEL	SLAC	FUBAR	
nsp1	96	32	268	268/535 (50.1)
nsp2	160	49	453	453/798 (56.8)
nsp3	124	53	280	281/530 (53.0)
nsp4	159	62	364	364/611 (59.6)
C	57	22	123	123/261 (47.1)
E3	17	5	30	30/64 (46.9)
E2	97	29	216	304/423 (71.9)
6 K	11	3	23	49/61 (80.3)
E1	98	37	244	293/439 (66.7)

*A site was considered under negative selection when detected by at least one of the following methods: FEL or SLAC at *p* ≤ 0.05, or FUBAR at posterior probability ≥ 0.95. SLAC, Single-Likelihood Ancestor Counting; FEL, Fixed Effects Likelihood; FUBAR, Fast Unconstrained Bayesian Approximation

## Discussion

Our outbreak investigation confirmed an active CHIKV outbreak in Kampung Baru Air Kuning in late 2019, which subsequently spread to the adjacent Bidor and Sungkai in early 2020. The high anti-CHIKV IgM positivity rate among the seropositive samples, together with observed IgM seroconversion, indicated active and recent CHIKV transmission rather than past infection [29]. The CHIKV seroprevalence in the present study (52.9%, 36/68) was comparable to the post-outbreak seroprevalence (recorded 3 months after the last reported case) in Tanjung Sepat, Selangor state, in August 2019 (72.5%, 108/149) [30]. DENV coinfection was also observed in this study, corroborating the dengue-hyperendemicity in Malaysia, where all four serotypes circulate persistently over time [31].

The ECSA-IOL strain has circulated globally since 2005 and was previously responsible for the 2008–2009 outbreak in Malaysia [9]. However, Malaysia 2019 and 2009 outbreak isolates were genetically distinct, forming separate branches in the phylogenetic tree, indicating an evolutionary shift over time. Instead, the Malaysia 2019 outbreak isolates clustered with the recent outbreak isolates from Thailand, China, Myanmar, Bangladesh, Italy, Pakistan and India during 2015–2019. The relatively higher nucleotide similarities among Malaysia 2019, China 2019 and Thailand 2018 outbreak isolates indicated the circulation of genetically similar CHIKV across multiple countries in the region.

The Thailand 2018 outbreak preceded the resurgence of CHIKV cases in Malaysia. Thailand reported a total of 3,580 cases in 2018, with an exponential increase in November (1,171 cases) and December (1,759 cases), whereas Malaysia recorded only 87 cases during the same year [32, 33]. Thailand is a popular tourist destination, receiving 39.92 million tourists in 2019, with Malaysia (10.7%) and China (27.9%) accounting for the largest proportions [34]. The high volume of tourism likely facilitated the cross-border transmission of CHIKV from Thailand into Malaysia and China. This is further supported by an internal case investigation report by Batang Padang District Health Office, which identified the first two Thailand imported cases as the index cases (unpublished data). Chronologically, the ECSA-IOL strain first reemerged in India in 2015 [35], subsequently spread to Pakistan [36] and Kenya [37] in 2016, to Bangladesh [38] and Italy [39] in 2017. Following that, it was imported from Bangladesh into Zhejiang Province, China in 2017 [40] and Thailand in 2018 [32]. Thereafter, CHIKV spread from Thailand to its neighbouring countries including Myanmar [41], China [42] and Malaysia in 2019.

The absence of a significant association between CHIKV positivity and sex (*p* = 0.943) or age group (*p* = 0.865) is consistent with findings from a systematic review on CHIKV in the Asia-Pacific region. This review analysed 52 records describing the age and sex distribution of CHIKV-affected individuals and found that the majority (69.2%) reported CHIKV infection across all age groups. Regarding sex, individual records varied in the sex predominance, with approximately equal proportions reporting male predominance (35%), female predominance (32%), and equal affectedness (30%) [43]. The absence of a consistent pattern across studies suggests that neither sex carries a substantially greater risk of CHIKV positivity. Among CHIKV-positive subjects, the lack of significant associations of sex or age with clinical manifestations indicates that the CHIKV clinical presentations are broadly consistent across demographic groups. However, the borderline association observed between age and rash (*p* = 0.060) may warrant further investigation in a larger cohort. Regarding dpo, rash was significantly more prevalent in CHIKV-positive subjects with ≥ 4 dpo (*p* = 0.024). In contrast, fever and joint pain were more prevalent in CHIKV-positive subjects with ≤ 3 dpo, though they did not reach statistical significance, likely reflecting the limited sample size. These findings on clinical symptom progression are consistent with a study reporting that subjects with fever and joint pain presented at a mean of 1.2 dpo, while rash emerged later at a mean of 3.5 dpo [44]. The early acute phase of CHIKV infection is characterised by fever and joint pain, while rash more frequently manifests in the later phase.

Selection pressure analysis identified only 26 positively selected sites across the protein-coding regions, with the majority under purifying or neutral selection. Unlike most other RNA viruses, arboviruses must replicate in both vertebrate and invertebrate hosts. This dual-host transmission cycle imposes strong evolutionary constraint, as mutations beneficial in one host may be detrimental in the other. Consequently, most mutations are likely removed by purifying selection, whereas neutral mutations may persist through genetic drift. This reflects an evolutionary trade-off between maintaining essential viral functions and allowing limited adaptive evolution [45, 46]. Among the 26 positively selected sites, only two (nsP3-258 and nsP3-372) correspond to amino acid substitutions between Malaysia 2009 and 2019 outbreak strains. As nsP3 plays a critical role in regulating non-structural polyprotein processing and viral replication, positive selection at these two nsP3 sites may have functional implications [47]. Further experimental validation is required to confirm their functional significance.

The Malaysia 2019 outbreak isolates harboured E1-226 A that was known to enhance viral fitness in *Ae. aegypti*, along with two epistatic mutations E1-K211E and E2-V264A that further increase vector adaptation when combined with E1-226 A [48]. These mutations were consistently observed in all recent outbreak isolates during 2015–2019. Notably, the Malaysian strain in 2020–2021 was also found to harbour these mutations [49], suggesting a sustained genetic adaptation toward *Ae. aegypti* during these recent epidemics. Further entomological study is required to identify the primary vector driving these epidemics. Additionally, Malaysia 2019 isolates and the majority of recent outbreak isolates harboured nsP3-R524Opal. This opal variant has been shown to be more virulent than the arginine variant in a mouse model, producing greater footpad swelling, more severe inflammation of ankle joints and tendons, and higher levels of proinflammatory cytokines and chemokines [50]. This raises concerns for potentially greater disease severity in infected patients. The other identified substitutions in the reemerging CHIKV could be potential epidemic-causing mutations but their impact on virulence and pathogenicity remains unclear and requires further studies. Continuous disease surveillance and molecular characterization of CHIKV are crucial for preventing and controlling future outbreaks, as well as understanding viral evolution and transmission dynamics.

The findings of this outbreak investigation were integrated into the local public health response. The laboratory diagnostic results were promptly reported to the local general practitioners and the Batang Padang District Health Office. These reports facilitated public health response in the affected hotspots, including intensified disease surveillance and vector control measures such as fogging. Furthermore, community health programs were conducted in collaboration with the District Health Office to increase public awareness of mosquito-borne infectious diseases. The local public health response at the study sites is detailed in Supplementary Fig. 1. Such collaborative efforts with the public health authorities are essential for the effective management and control of reemerging outbreaks.

## Conclusion

The reemerging ECSA-IOL strain in Malaysia after a decade of sporadic transmission was genetically distinct from the strain responsible for the previous 2009 outbreak in Malaysia, but closely related to the globally circulating epidemic strains during 2015–2019. Collectively, our findings suggested that the reemerging CHIKV in Malaysia was likely introduced from neighbouring Thailand through cross-border transmission. Robust surveillance strategies and cross-border collaboration are important for early detection and control of future outbreaks.

## Supplementary Information

Below is the link to the electronic supplementary material.

Supplementary Material 1

Supplementary Material 2

## Data Availability

The CHIKV sequence data generated in this study have been deposited in the NCBI GenBank database under the following accession numbers: PX569501, PX569502, PX569503, and PX569504.

## Abbreviations

BLAST: Basic Local Alignment Search Tool
C: Capsid protein
CDS: Complete coding sequences
CHIKV: Chikungunya virus
COL: Caribbean Outbreak Lineage
DENV: Dengue virus
EMEM: Eagle’s Minimum Essential Medium
FEL: Fixed Effects Likelihood
FUBAR: Fast Unconstrained Bayesian Approximation
GSNAP: Genomic Short-read Nucleotide Alignment Program
dpo: Days post-onset
E: Envelope protein
ECSA: East/Central/South African
ELISA: Enzyme-linked immunosorbent assay
FBS: Fetal bovine serum
IOL: Indian Ocean Lineage
IQR: Interquartile range
MEGA: Molecular Evolutionary Genetics Analysis
MEME: Mixed Effects Model of Evolution
ML: Maximum likelihood
NJ: Neighbour joining
nsP: Non-structural protein
ORF: Open reading frame
RT-PCR: Reverse transcription polymerase chain reaction
SLAC: Single-Likelihood Ancestor Counting
SPSS: Statistical Package for Social Sciences
TIDREC: Tropical Infectious Diseases Research and Education Centre
ZIKV: Zika virus
6K: Accessory protein
