# Supplementary material for: Outbreak investigation of reemerging chikungunya virus in Malaysia in 2019–2020
Source: BMC Infect Dis. 2026 Jun 11;26:1522. doi: 10.1186/s12879-026-13793-7 (PMC13488065; doi:10.1186/s12879-026-13793-7)
Supplement: Supplementary file 2 [file 12879_2026_13793_MOESM2_ESM.docx]

Supplementary Table 1 Primers and probes used in real-time RT-PCR assays for CHIKV, DENV and ZIKV detection*

| **Target virus** | **Oligo type** | **Nucleotide sequences (5’🡪 3’)** | **Amplicon size (bp)** | **Target gene** |
| --- | --- | --- | --- | --- |
| CHIKV | F | GCAGARGAAGAACGAGAAGC | 174 | *nsP1–nsP2* |
|  | R | GTGGTCYGTTRGTTGGGCAG |  |  |
|  | P | [FAM]-CGAAATTGACGTGGAAC-[MGB- NFQ] |  |  |
|  |  |  |  |  |
| DENV | F | ACCATGGAAGCTGTACGC | 219 | 3’ UTR |
|  | R | CGTTCAGTGCCTGGATTGATG |  |  |
|  | P | [FAM]-CAGAGATCCTGCTGTCT-[MGB- NFQ] |  |  |
|  |  |  |  |  |
| ZIKV | F | GGCACTAGATGAGAGTGGTGA | 110 | *NS2B* |
|  | R | CTATTGGGTTCATGCCACAGA |  |  |
|  | P | [FAM]-AGAGAGATCATACTCAAGG-[MGB-NFQ] |  |  |

*CHIKV, chikungunya virus; DENV, dengue virus; F, forward primer; MGB-NFQ, minor groove binder non-fluorescent quencher; P, TaqMan probe; R, reverse primer; RT-PCR, reverse transcription polymerase chain reaction; ZIKV, Zika virus.
