# Supplementary material for: Outbreak investigation of reemerging chikungunya virus in Malaysia in 2019–2020
Source: BMC Infect Dis. 2026 Jun 11;26:1522. doi: 10.1186/s12879-026-13793-7 (PMC13488065; doi:10.1186/s12879-026-13793-7)
Supplement: Supplementary file 3 [file 12879_2026_13793_MOESM1_ESM.docx]

Supplementary Fig. 1 Local public health response at study sites in Perak, 2019–2020.
